# Universalismo Privatizante no sistema de saúde brasileiro e o Programa Agora Tem Especialistas

**DOI:** 10.1590/0102-311XPT244725

**Published:** 2026-10-05

**Authors:** Danielle Conte Alves Riani Costa, Ligia Bahia, José Felipe Riani Costa, José Luiz Riani Costa

**Affiliations:** 1 Agência Nacional de Saúde Suplementar, Rio de Janeiro, Brasil.; 2 Instituto de Estudos em Saúde Coletiva, Universidade Federal do Rio de Janeiro, Rio de Janeiro, Brasil.; 3 Claretiano - Centro Universitário de Rio Claro, Rio Claro, Brasil.

**Keywords:** Sistemas de Saúde, Sistema Único de Saúde, Saúde Suplementar, Política de Saúde, Financiamento da Assistência à Saúde, Health Systems, Unified Health System, Supplemental Health, Health Policy, Healthcare Financing, Sistemas de Salud, Sistema Único de Salud, Salud Complementaria, Política de Salud, Financiación de la Atención de la Salud

## Abstract

O artigo busca refletir sobre o Programa Agora Tem Especialistas (PATE) à luz do conceito de Universalismo Privatizante no sistema de saúde brasileiro. O PATE, divulgado pelo Ministério da Saúde, visa a ampliar a capacidade de atendimento e reduzir a fila de pacientes do Sistema Único de Saúde (SUS). Considerando-se as políticas de saúde introduzidas pelo PATE, ao envolver os planos de saúde e os hospitais privados, é fundamental fazer uma análise deste Programa com base no referencial teórico sobre o Universalismo Privatizante, que envolve uma avaliação da relação entre o público e o privado no sistema de saúde brasileiro. O conceito diz respeito à forma como a universalidade vem sendo alcançada, por meio da universalização do componente privado. São apontadas as políticas trazidas pelo Programa, bem como os principais desafios. Constatou-se que o PATE não deve ser considerado uma dimensão do Universalismo Privatizante por não envolver a elevação do gasto privado com saúde, não gerar isenções fiscais, não promover a expansão dos planos de saúde, nem a transferência das funções e responsabilidades do setor público para o privado. Além disso, busca mais recursos para o SUS, a redução das disparidades no acesso aos serviços de saúde e coloca o setor privado de saúde a serviço da população brasileira. Torna-se imprescindível monitorar as repercussões do PATE e os seus resultados na redução das desigualdades em saúde, principalmente no que se refere às discrepâncias atualmente existentes na distribuição dos recursos e na utilização de serviços entre as regiões do país e entre os segmentos público e privado de saúde.

## Abstract (en)

This article reflects on the Now There Are Specialists Program (PATE, acronym in Portuguese) in light of the concept of Privatizing Universalism in the Brazilian health system. The program, presented by the Brazilian Ministry of Health, aims to expand its capacity to provide care and reduce patient waiting lists in the Brazilian Unified National Health System (SUS, acronym in Portuguese). Given the health policies introduced under the PATE, which involve health insurance plans and private hospitals, it is essential to analyze the Program based on the theoretical framework of Privatizing Universalism, which assesses the relationship between the public and private sectors in the Brazilian health system. The concept refers to the way universality has been pursued, through the universalization of the private component. The article identifies the policies introduced by the PATE, as well as the main challenges. The study found that PATE should not be considered a dimension of Privatizing Universalism because it does not increase private health spending, generate tax exemptions, promote the expansion of health insurance plans, or transfer functions and responsibilities from the public to the private sector. In addition, it seeks to secure more resources for the SUS, reduce disparities in access to health services, and place the private health sector at the service of the Brazilian population. It is essential to monitor the repercussions of the PATE and its results in reducing health inequalities, especially regarding the discrepancies that currently exist in the distribution of resources and in the use of services between the country’s regions and between the public and private health sectors.

## Abstract (es)

El artículo busca reflexionar sobre el Programa Ahora Hay Especialistas (PATE, por su sigla en portugués) a la luz del concepto de Universalismo Privatizador en el sistema de salud brasileño. El PATE, dado a conocer por el Ministerio de Salud, tiene como objetivo ampliar la capacidad de atención y reducir la lista de espera de pacientes del Sistema Único de Salud (SUS). Teniendo en cuenta las políticas de salud introducidas por el PATE, al involucrar a los planes de salud y a los hospitales privados, es imprescindible realizar un análisis de este Programa basándose en el marco teórico del Universalismo Privatizador, lo cual implica una evaluación de la relación entre lo público y lo privado en el sistema de salud brasileño. El concepto se refiere a la forma en que se ha ido logrando la universalidad, a través de la universalización del componente privado. Se destacan las políticas que introduce el PATE, así como los principales desafíos. Se constató que el PATE no debe considerarse una dimensión del Universalismo Privatizador, ya que no implica un aumento del gasto privado en salud, no genera exenciones fiscales, no promueve la expansión de los planes de salud ni la transferencia de funciones y responsabilidades del sector público al privado. Además, busca más recursos para el SUS, la reducción de las disparidades en el acceso a los servicios de salud y pone al sector privado de la salud al servicio de la población brasileña. Es imprescindible monitorear las repercusiones del PATE y sus resultados en la reducción de las desigualdades en materia de salud, especialmente en lo que se refiere a las discrepancias que existen actualmente en la distribución de los recursos y en el uso de los servicios entre las regiones del país y entre los segmentos público y privado de la salud.

## Introdução

O presente texto busca refletir sobre o Programa Agora Tem Especialistas (PATE) à luz do conceito de Universalismo Privatizante no sistema de saúde brasileiro.

O PATE, divulgado pelo Ministério da Saúde, visa a ampliar a capacidade de absorver a demanda e reduzir a fila de pacientes do Sistema Único de Saúde (SUS) à espera de atendimento, por meio de uma série de medidas, como a utilização da rede prestadora do setor privado, incluindo os planos de saúde.

Cabe destacar que, além da parceria com a rede privada, o PATE prevê como linhas de ação: formação e provimento de especialistas; telessaúde; unidades móveis; rede de diagnóstico de câncer; mutirões para acelerar atendimentos; painel nacional para monitorar fila; e o aplicativo Meu SUS Digital ^1^.

No caso da parceria, clínicas e hospitais com ou sem fins lucrativos podem ser credenciados para realizar consultas, exames e cirurgias pelo SUS, em troca de créditos ou dívidas tributárias dos estabelecimentos com a União. A parceria com os planos de saúde prevê o abatimento das dívidas com o ressarcimento ao SUS ^1^.

As desigualdades na oferta e produção de serviços de saúde são conhecidas, tanto em termos de distribuição dos serviços entre as regiões do país, quanto no que se refere à concentração de recursos no setor privado ^2^ ^,^ ^3^ ^,^ ^4^ ^,^ ^5^.

Com o lançamento do Programa, uma dúvida foi instantânea: será o PATE mais uma dimensão do Universalismo Privatizante no nosso sistema de saúde?

Considerando as políticas de saúde introduzidas pelo Programa, ao envolver os planos de saúde e os hospitais privados, é fundamental fazer uma análise do PATE à luz do referencial teórico sobre o Universalismo Privatizante, que envolve uma análise da relação entre o público e o privado no sistema de saúde brasileiro.

## O Universalismo Privatizante

O conceito de Universalismo Privatizante diz respeito à forma como a universalidade vem sendo alcançada no sistema de saúde brasileiro, por meio da universalização do componente privado. As dimensões de análise utilizadas para fundamentar o conceito estão sistematizadas na Figura 1 ^5^.

**Figura 1:**
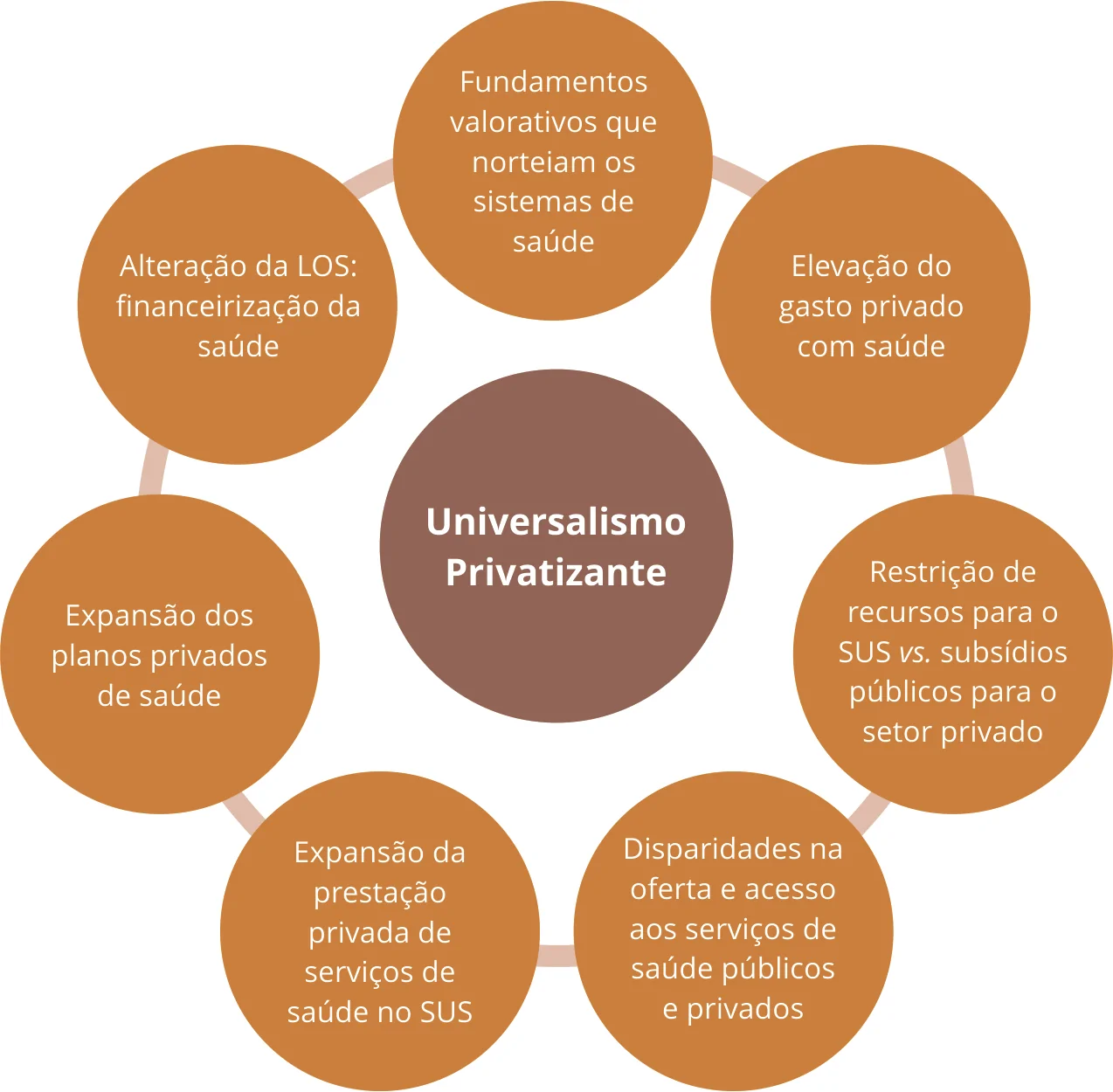
Diagrama do Universalismo Privatizante no sistema de saúde brasileiro.

LOS: Lei Orgânica da Saúde; SUS: Sistema Único de Saúde. Fonte: adaptado de Costa & Bahia ^5^.

Os sete eixos que compuseram a análise do Universalismo Privatizante, detalhados no livro *Crise Econômica e Sanitária e o Universalismo Privatizante no Sistema de Saúde do Brasil* ^5^, permitem identificar como o movimento de universalização do setor privado se expressa no sistema de saúde brasileiro, tanto pelo fomento à ampliação do setor suplementar, por meio de subsídios e políticas públicas, quanto pela gestão e pela prestação privada de serviços no âmbito do próprio SUS. Esse panorama é complementado pela restrição de recursos financeiros para a saúde pública.

## Novas políticas de saúde: a mudança do paradigma no sistema de saúde brasileiro

É possível identificar, a partir de 2003, medidas do Ministério da Saúde para ampliar o financiamento federal da atenção especializada (AE) ^6^. Em 2023, foi instituída a primeira Política Nacional de Atenção Especializada em Saúde (PNAES) contemplando aspectos como estratégias de formação, saúde digital e Ofertas de Cuidados Integrados (OCI) ^7^, os quais estão presentes em componentes do PATE.

O Programa Mais Acesso a Especialistas (PMAE), instituído em 2024, aprofundou o conceito e a relevância das OCI na organização e no monitoramento das ações e serviços de saúde ^8^.

Desde o seu lançamento, em maio de 2025, por meio da *Medida Provisória nº 1.301*, o PATE, criado a partir da unificação do Programa Nacional de Redução de Filas e do PMAE, agregou novos elementos e gerou diversas publicações entre normativos e manuais técnicos, além de webinários, o que, para fins de pesquisa, envolveu a busca de muitos documentos e a necessidade de encaixar algumas peças do quebra-cabeça para a compreensão desta nova política de saúde. A sanção da *Lei nº 15.233*, de 7 de outubro de 2025, garantiu a vigência do PATE até 2030 ^9^.

O PATE mantém o conjunto de estratégias do PMAE e agrega novos dispositivos, como o componente cirurgia e a ampliação da participação do setor privado na execução de serviços, incluindo mecanismos de compensação de dívidas e de ressarcimento ao SUS. Dessa forma, o PATE reconhece a importância da transversalidade da saúde e a relação com a economia ao criar incentivo financeiro para aumentar a realização de procedimentos no SUS; e com a educação, por conta do Projeto Mais Médicos Especialistas, demonstrando a importância da articulação intersetorial para a efetivação de políticas de saúde.

Com o estabelecimento da PNAES e o advento do PATE foram delineadas as diretrizes e definidas as estratégias para o enfrentamento dos principais desafios da AE, com base em uma agenda tripartite, que configura o ineditismo da formulação de políticas de AE para o SUS. Essa agenda muda o paradigma das políticas de saúde no Brasil ao proceder à maior reforma efetuada nos últimos anos.

### AE e acesso como prioridades

Durante muitos anos, as políticas públicas de saúde focaram no fortalecimento da atenção básica (AB), o que permitiu um aumento da cobertura da atenção primária à saúde (APS), especialmente por meio da Estratégia Saúde da Família (ESF), permitindo o acesso a um grande contingente da população. Com isso, muitos casos que estavam fora do sistema de saúde ganharam visibilidade, evidenciando a insuficiência da AE.

Sem diminuir a importância da AB, o PATE muda o paradigma ao consolidar o ineditismo da formulação de políticas de AE como prioridade das políticas de saúde no país, fato de suma relevância diante da dificuldade de atendimento, da transição epidemiológica e da necessidade de enfrentamento das doenças crônicas não transmissíveis (DCNT).

As especialidades prioritárias do PATE, definidas pelo PMAE no âmbito da PNAES, são: oncologia, cardiologia, ortopedia, otorrinolaringologia e oftalmologia.

Destaca-se que o PATE visa à redução das filas e à garantia de atendimento em locais de difícil acesso, priorizando regiões de vazios assistenciais.

### Créditos ou dívidas tributárias de estabelecimentos

Clínicas e hospitais com ou sem fins lucrativos podem ser credenciados para realizar atendimentos pelo SUS. Em compensação, ocorre a troca de créditos ou dívidas tributárias dos estabelecimentos com a União por prestação de atendimentos especializados, ou seja, em vez de receber por valores de atendimento, os estabelecimentos acumulam créditos financeiros que podem ser usados para compensar dívidas existentes com o governo ou aplicados na quitação de futuros pagamentos de tributos.

No caso de pagamento das dívidas, os valores são direcionados para o Tesouro Nacional e irão compor o Orçamento Geral da União. Nesse sentido, o PATE angaria mais recursos para a saúde ao garantir que os valores que iriam para a União possam ser diretamente investidos no atendimento à saúde da população.

A despeito do avanço trazido pelo fim da *Emenda Constitucional 95* (EC 95), o novo arcabouço fiscal retomou o limite de 15% da Receita Corrente Líquida da EC 86 ^10^, ou seja, o orçamento depende da capacidade de crescimento da economia. Nesse sentido, a possibilidade de reverter créditos e dívidas tributárias para a realização de procedimentos de AE no SUS representa uma ampliação de recursos para o orçamento do Ministério da Saúde.

Vale ressaltar que a Lei do PATE define que os créditos financeiros estão limitados anualmente ao valor de 2 bilhões de Reais, o que significa que existe um teto para o abatimento de débitos tributários ^9^.

### Planos de saúde e ressarcimento ao SUS

O PATE estabelece a possibilidade de empresas de planos de saúde se habilitarem ao Programa e abaterem suas dívidas com o ressarcimento ao SUS.

O ressarcimento ao SUS foi criado pela *Lei nº 9.656/1998*, a qual determina que as operadoras devem ressarcir os gastos sempre que um beneficiário de plano de saúde for atendido no SUS ^11^.

O valor ressarcido ao erário é depositado no Fundo Nacional de Saúde (FNS). Esse valor é direcionado ao financiamento das Ações e Serviços Públicos de Saúde (ASPS). O PATE cria um mecanismo que redireciona a destinação desses valores direto para a realização de ASPS. Dessa forma, por uma via ou por outra, os valores de ressarcimento são direcionados à prestação de serviços no SUS.

É importante ressaltar que essas dívidas demoram para ser pagas, algumas empresas não pagam ou entram na justiça. Dados publicados pela Agência Nacional de Saúde Suplementar (ANS), em junho de 2025, demonstraram que, do total de R$ 16,3 bilhões em dívidas de operadoras com o ressarcimento ao SUS identificadas entre 1999 e 2024, somente R$ 7,5 bilhões haviam sido efetivamente pagas ou estavam em parcelamento ^12^.

Ao possibilitar o redirecionamento desses valores diretamente para garantir a realização de atendimentos especializados no SUS, o PATE alinha a necessidade de elaborar políticas para dirimir as filas existentes e a garantia de recebimento dos valores cobrados pelo ressarcimento.

### Regulação das filas

O acompanhamento da execução e controle dos atendimentos realizados no âmbito do PATE será feito pelos sistemas de regulação. O Sistema Nacional de Regulação (SISREG), utilizado na regulação das filas, está sendo substituído pelo e-SUS Regulação, sistema que busca integrar os dados nacionais. O módulo e-SUS captação de filas será voltado para locais sem sistema informatizado de regulação.

A regulação das filas é uma das maiores fragilidades do PATE, pois não foi instituído um processo sistemático para o monitoramento do acesso e da quantidade de pessoas aguardando para realizar procedimentos.

### Sistemas de informação

O PATE previu a criação de habilitações exclusivas no Cadastro Nacional de Estabelecimentos de Saúde (CNES), a fim de cadastrar a rede assistencial prestadora que adere ao Programa ^13^, e a efetivação de adequações nos sistemas de informação para o lançamento da produção assistencial relacionada ao PATE no Sistema de Informações Ambulatoriais (SIA) e no Sistema de Informações Hospitalares (SIH).

Existe a previsão de substituir esses sistemas pelo Conjunto Mínimo de Dados da Atenção à Saúde (CMD), documento público que coletará e padronizará os dados de todos os atendimentos de saúde no Brasil, realizados em estabelecimentos públicos e privados. O CMD, que já recebe dados assistenciais do Sistema de Informação da Atenção Básica (SIAB) e da saúde suplementar, é integrado à Rede Nacional de Dados em Saúde (RNDS) ^13^.

## Hospitais privados e planos de saúde prestando serviços para os usuários do SUS: inovação ou mais uma dimensão do Universalismo Privatizante?

A prática da contratação de serviços em hospitais privados para a prestação em unidades públicas sempre foi concebida no SUS desde a sua instituição. O que mudou para esse segmento foi a possibilidade que o PATE trouxe de abater dívidas tributárias ou originar créditos financeiros, conforme exposto em subitem anterior.

A novidade do PATE nesse quesito é a possibilidade de introduzir no SUS os hospitais eminentemente privados que antes não prestavam atendimento aos usuários do SUS, e as empresas de planos de saúde, que podem prestar atendimento por meio da sua rede verticalizada ou credenciada. A adesão das operadoras de planos de saúde garante a devolução, ao FNS, dos valores de ressarcimento devidos ao SUS.

Costa & Bahia ^5^ apontam que o setor privado de saúde no Brasil recebe isenções fiscais de várias formas, no entanto, não gera nenhuma contrapartida para a população. O PATE inova ao integrar o SUS e o setor privado de saúde, ao permitir abatimento de dívidas e geração de créditos financeiros e, principalmente, ao gerar uma contrapartida para a população.

Cabe recordar que a proposta de fila única para acesso a leitos de unidades de tratamento intensivo (UTI) foi refutada na pandemia de COVID-19. Enormes filas no SUS enquanto os hospitais privados tinham leitos ociosos ^3^.

O PATE pode ser considerado uma iniciativa que mobiliza recursos assistenciais de hospitais privados e empresas de planos de saúde em prol da garantia de atendimento especializado aos usuários do SUS, a fim de ampliar o acesso aos serviços de saúde. Para tanto, angaria recursos para aumentar a capacidade de atendimento do SUS e convoca o setor privado para esta missão.

Além das questões supramencionadas, o PATE não deve ser considerado uma dimensão do Universalismo Privatizante por não envolver a elevação do gasto privado com saúde, não gerar isenções fiscais, não promover a expansão dos planos de saúde, nem a transferência das funções e responsabilidades do setor público para o privado. Além disso, busca mais recursos para o SUS e redução das disparidades no acesso aos serviços de saúde.

Pode-se mencionar, ainda, que o PATE não corrobora a necessidade de investir nos planos de saúde para “desafogar” o SUS e coloca o setor privado de saúde a serviço da população brasileira.

## Desafios

Questões fundamentais não foram abordadas pelo PATE, como: Quais serão os desfechos? Quais são as metas em termos de melhoria da saúde da população?

O PATE está direcionado ao aumento da produção de serviços, mas não explicita as estratégias para o aumento efetivo e sustentável da capacidade instalada, da oferta universal de serviços de saúde e do orçamento do SUS.

Para além de mobilizar recursos financeiros frente às limitações no orçamento da saúde, o PATE precisará avançar na proposição de políticas para expandir o financiamento e a oferta de serviços públicos de saúde, movimento necessário no sentido de fortalecer e assegurar as bases de sustentação da política e diminuir as desigualdades em saúde.

Além disso, a entrada do setor privado no PATE não corrige as iniquidades regionais em função da concentração da oferta privada nas regiões onde existe maior oferta de serviços públicos e privados contratualizados pelo SUS.

A ampliação da rede assistencial é crucial para que, de fato, ocorram impactos perenes na oferta e acesso equânime aos cuidados de saúde.

É fundamental de manter o fortalecimento da AB e de qualificar a APS por meio do apoio especializado matricial ^14^ ^,^ ^15^, principalmente, nas áreas prioritárias estabelecidas pelo PATE. Tal metodologia envolve a organização dos fluxos na Rede de Atenção à Saúde, rompendo a burocratização, a inclusão de profissionais da saúde de diferentes áreas de forma integrativa, e a garantia de recursos materiais.

Convém incluir equipes de saúde multidisciplinares e investir na sua formação e educação permanente, assim como fortalecer as ações de promoção da saúde e prevenção de doenças, com ênfase em alimentação saudável, prática de atividade física, saúde mental, controle de tabagismo e alcoolismo, fatores reconhecidamente relacionados às DCNT que geram grande demanda para a AE.

Em comparação com a APS e com a atenção hospitalar, a AE configura uma das áreas menos estudadas. Com o advento dos investimentos no PATE, ganha maior relevância a necessidade de avaliar o PATE, incluindo a sua estruturação, pressupostos e possíveis impactos ^16^ ^,^ ^17^.

Torna-se imprescindível dar transparência às filas, monitorar as repercussões do PATE e seus resultados na redução das desigualdades em saúde, principalmente no que se refere às discrepâncias atualmente existentes na distribuição dos recursos e na utilização de serviços entre as regiões do país e entre os segmentos público e privado de saúde.

## Data Availability

Não se aplica.

## References

[1] Ministério da Saúde Agora Tem Especialistas..

[2] Paim JS, Travassos C, Almeida C, Bahia L, Macinko J (2011). O sistema de saúde brasileiro história, avanços e desafios. Lancet.

[3] Costa DCAR, Bahia L, Carvalho EMCL, Monte-Cardoso A, Souza PMS (2020). Oferta pública e privada de leitos e acesso aos cuidados à saúde na pandemia de COVID-19 no Brasil.. Saúde Debate.

[4] Costa DCAR, Moreira JPL, Cardoso AM, Mattos LV, Andrietta LS, Bahia L (2022). Crise econômica e disparidades no gasto, oferta e utilização de serviços públicos e privados de saúde no Brasil no período entre 2011 e 2019. Cad Saúde Pública.

[5] Costa DCAR, Bahia L (2024). Crise econômica e sanitária e o universalismo privatizante no sistema de saúde do Brasil.

[6] Solla J, Chioro A, Giovanella L, Escorel S, Lobato LVC, Noronha JC, Carvalho AI (2012). Políticas e sistemas de saúde no Brasil..

[7] Ministério da Saúde (2023). Portaria GM/MS nº 1.604, de 18 de outubro de 2023. Institui a Política Nacional de Atenção Especializada em Saúde (PNAES), no âmbito do Sistema Único de Saúde.. Diário Oficial da União.

[8] Ministério da Saúde (2024). Portaria GM/MS nº 3.492, de 8 de abril de 2024. Institui o Programa Nacional de Expansão e Qualificação da Atenção Ambulatorial Especializada, no âmbito do Sistema Único de Saúde (SUS).. Diário Oficial da União.

[9] Brasil (2025). Lei nº 15.233, de 7 de outubro de 2025. Institui o Programa Agora Tem Especialistas; dispõe sobre o Grupo Hospitalar Conceição S.A.; altera as Leis nºs 8.080, de 19 de setembro de 1990 (Lei Orgânica da Saúde), 8.958, de 20 de dezembro de 1994, 9.656, de 3 de junho de 1998, 12.732, de 22 de novembro de 2012, 12.871, de 22 de outubro de 2013, e 13.958, de 18 de dezembro de 2019.. Diário Oficial da União.

[10] Ocké-Reis CO, Benevides R, Funcia F, Melo M (2023). Evolução do piso federal em saúde: 2013-2020.

[11] Brasil (1998). Lei nº 9.656, de 3 de junho de 1998. Dispõe sobre os planos e seguros privados de assistência à saúde.. Diário Oficial da União.

[12] Agência Nacional de Saúde Suplementar Panorama do ressarcimento ao SUS..

[13] Coordenação-Geral de Gestão de Sistemas de Informação em Saúde. Departamento de Regulação Assistencial e Controle. Secretaria de Atenção Especializada à Saúde. Ministério da Saúde (2025). Nota Informativa nº 125/2025-CGSI/DRAC/SAES/MS.

[14] Campos GWS (1999). Equipes de referência e apoio especializado matricial uma proposta de reorganização do trabalho em saúde. Ciênc Saúde Colet.

[15] Campos GWS, Domitti AC (2007). Apoio matricial e equipe de referência uma metodologia para gestão do trabalho interdisciplinar em saúde. Cad Saúde Pública.

[16] Conselho Nacional de Secretários de Saúde (2016). Debate - Inovação na Atenção Ambulatorial Especializada.

[17] Silva SM (2023). Avaliação de políticas e programas nacionais de saúde do Brasil: uma revisão de escopo.

